# Proposition of a simple binary grading of estimated blood loss during colon surgery

**DOI:** 10.1007/s00384-021-03925-7

**Published:** 2021-04-16

**Authors:** Hugo Teixeira Farinha, David Martin, Audrey Ramó, Martin Hübner, Nicolas Demartines, Dieter Hahnloser

**Affiliations:** Department of Visceral Surgery, University Hospital Lausanne, CHUV, Rue du Bugnon 46, 1011 Lausanne, Switzerland

**Keywords:** Estimation, Blood loss, Colon surgery, Post-operative complications

## Abstract

**Purpose:**

Intraoperative estimated blood loss (EBL) is often reported in nearly all surgical papers; however, there is no consensus regarding its measurement. The aim of this study was to determine whether EBL (ml) is as reliable and reproducible in predicting complications as a simple binary grading of EBL.

**Methods:**

All consecutive patients undergoing colectomies between January 2015 and December 2018 were included. EBL was assessed prospectively by the surgeon and anaesthesiologist in ml and with a binary scale: bleeding “as usual” versus “more than usual” by the surgeon. Differences between pre- and post-operative haemoglobin levels (ΔHb g/dl) were correlated to EBL. Blood loss impact on 30-day postoperative morbidity was analysed.

**Results:**

A total of 270 patients were included, with a mean age of 65 years (SD 17). Mean EBL documented by surgeons correlated to EBL by anaesthesiologists (79.5 ml, SD 99 vs. 84.5 ml, SD 118, ϱ = 0.926, *p* < 0.001). Surgeons and anaesthesiologists’ EBL correlated also with ΔHb (ϱ = − 0.273, *p* = 0.01 and *ϱ* = − 0.344, *p* = 0.01, respectively). Patient with surgeon EBL ≥ 250 ml or graded as “more than usual” bleeding had significantly more severe complications (8% vs. 20%, *p* = 0.02 and 8% vs. 27%, *p* = 0.001, respectively).

**Conclusion:**

Anaesthesiologist and surgeon’s EBL correlated with ΔHb. Simple grading of blood loss as “usual” and “more than usual” predicted severe complications and higher mortality rates. This simple binary grading of blood loss in colon surgery could be an alternative to the estimation of blood loss in ml as it is easy to apply but needs to be validated externally.

## Introduction

Estimated blood loss (EBL) is often reported on routine basis and is in many studies a risk factor for short- and long-term complications after colorectal surgery [1–7]. In a multicentre study including 1421 patients, EBL was associated with postoperative morbidity [1]. Two studies showed that EBL was an independent risk factor of prolonged postoperative ileus, however without clear cut-off in volume [2, 3]. Several retrospective studies showed that EBL ≥ 250 ml was associated with higher anastomotic leakage rate and 6-month mortality after colorectal surgery [4, 6]. In emergency colorectal procedures, high EBL (≥ 1000 ml) was associated with in-hospital mortality [5]. Furthermore, a review showed that intraoperative blood transfusions represented an independent risk factors for intra- and post-operative complications and adversely affected outcomes in colorectal surgery [7]. However, estimation of EBL remains subjective with no consensus on how to estimate it. Furthermore, data on accuracy of EBL are lacking.

Therefore, the aim of this study was to determine whether a simple binary grading of EBL in colon surgery is a reliable and reproducible measure in daily practice.

## Methods

### Patients

This study included all consecutive patients undergoing open and laparoscopic colectomies performed in elective and emergency settings between January 2015 and December 2018 in the Visceral Surgery Department at the University Hospital of Lausanne, Switzerland. Colon surgery included right colectomy (including ileocecal resections), extended right colectomy, transverse colectomy, left colectomy, segmental colectomy, sigmoidectomy (including Hartmann procedure) and total colectomy with or without anastomosis. Rectal resections were excluded from this analysis. Patients with other additional intra-abdominal procedures were excluded as well as patients under 18 years old.

### EBL measurements

EBL was prospectively documented by the surgeon and the anaesthesiologist in volume (ml) directly after the intervention. Surgeon EBL was entered in the institutional interactive software (Digistat®) used for the scheduling and the real-time vision of the operating theatre activity and based on visual estimation. Anaesthesiologist EBL was entered in the patient electronic file and assessed on the basis of the aspirated volume by deducting the lavage volume. The 2 specialists entered the data separately without consulting each other. Binary EBL defined “as usual” vs. “more than usual” was entered by the operating surgeon in the electronic patient file at the end of the operation, and based on subjective feelings regarding intraoperative bleeding, without any other predefined criteria or evaluation scale.

According to several studies [4, 6], clinically relevant EBL cut-off was set at 250 ml, which represents a packed red blood cells. Impact of EBL on 30-day postoperative morbidity was analysed.

### Data extraction

Primary extraction was performed from Digistat® using the key words: colectomy, hemicolectomy, sigmoidectomy, Hartmann procedure. Data included type of intervention, indication for surgery, surgeon EBL (ml), operating time (minutes), emergency degree and surgeon expertise (staff surgeon or consultant).

Other data of interest were collected from the institutional electronic patient file. Demographics included age, gender, BMI (kg/m^2^), malignancy, comorbidities and ASA score. Postoperative data were retrieved from the Enhanced Recovery After Surgery (ERAS) database and included length of stay, intensive care unit (ICU) stay, 30-day postoperative complications and mortality. Overall complications have been graded according to Clavien classification [8]. Severe complications were defined as grade ≥ 3b. Only the highest grade was retained in patients presenting more than one complication.

Differences between pre- and post-operative haemoglobin levels (ΔHb g/dl) were documented, as well as platelet count and coagulation parameters. Haemoglobin levels were systematically measured on the day before surgery and on postoperative day 1.

### Statistics

Continuous variables were presented as mean with standard deviation (SD) or median with interquartile range (IQR) according to their distribution. Categorical variables were reported as frequencies (%) and compared with chi-square test. Student’s *t* test or Mann–Whitney test were used for continuous variable comparisons. Statistical correlations between surgeon and anaesthesiologist EBL, as well as between surgeon EBL and ΔHb, were measured using Spearman’s rank correlation coefficient. All statistical tests were two-sided and a level of 0.05 was used to indicate statistical significance. Statistical analyses were performed with GraphPad Prism 8 (GraphPad Software, Inc., La Jolla, CA, USA).

### Ethics

The study was approved by local Commission on Ethics in Human Research (CER-VD, protocol number 2018-0280) and was conducted in compliance with the current version of the Declaration of Helsinki.

## Results

Three hundred and fourteen patients were assessed, of which 15 (4.8%) were excluded due to an objection to the use of their data for research, and an additional 29 (9.2%) were excluded due to the absence of documented surgeon and anaesthesiologist EBL. The present study thus included 270 patients.

Demographics, operative indication, malignancy, main comorbidities and laboratory values regarding surgeon EBL (< 250 vs. ≥ 250ml) are presented in Table 1. Surgeon EBL ≥ 250 ml was significantly associated with more emergency procedures.

**Table 1:** Baseline demographics, comorbidities and diagnosis: comparison between surgeon EBL < 250 ml vs ≥ 250 ml

Malignant	135 (50%)	118 (51%)	27 (68%)	
Hematochezia as main indication	10 (4%)	8 (3%)	2 (5%)	0.6
Urgency of surgery				**< 0.01**
Elective	196 (73%)	174 (76%)	22 (55%)	
Emergency	74(27%)	56 (24%)	18 (45%)	
ASA score				0.4
1	25 (9%)	21 (9%)	4 (10%)	
2	134 (50%)	116 (50%)	18 (45%)	
3	94 (35%)	79 (34%)	15 (38%)	
4	17 (6%)	14 (7%)	3 (7%)	
Preoperative coagulation parameters				
PT < 60%	15 (6%)	13 (6%)	2 (5%)	0.8
Thrombocytes <50 G/l	9 (3%)	8 (3%)	1 (3%)	0.8

Mean (*SD* standard deviation) or number (%) as appropriate. Statistical significance (*p* < 0.05) is highlighted in bold

*ASA* American Association of Anaesthesiologists physical status classification system, *PT* prothrombine time

Surgical details are shown in Table 2. Patients with surgeon EBL ≥ 250 ml had significantly more open procedures, longer surgical duration and more intraoperative transfusions. A significant drop in haemoglobin level (ΔHb) has been observed in patients with surgeon EBL ≥ 250 ml (− 20 vs − 12.5 g/l, *p* = 0.04). Sixteen patients had an intraoperative transfusion (5.9%).

**Table 2:** Surgical details: comparison between surgeon EBL < 250 ml vs ≥ 250 ml

	Total *n* = 270	EBL < 250 ml *n* = 230	EBL ≥ 250 ml *n* = 40	*p* value
Approach				**< 0.01**
Laparoscopy	186 (69%)	171 (74%)	15 (38%)	
Open	84 (31%)	59 (26%)	25 (62%)	
Mean surgical time (min; IQR)	149 (71.2)	146 (53.9)	201 (72.2)	**< 0.01**
< 2 h	27 (28%)	74 (32%)	2 (5%)	**< 0.01**
> 2 h	194 (72%)	156 (68%)	38 (95%)	
Surgeon				0.2
Junior staff	144 (53%)	119 (52%)	25 (63%)	
Senior staff	126 (47%)	111 (48%)	15(37%)	
Operation during night shift	32 (12%)	24 (10%)	8 (20%)	0.1
Intra-operative transfusion (*n* patients)	16 (6%)	6 (3%)	10 (25%)	**< 0.01**
Mean ΔHb (g/dl; SD)	− 1.4 (1.6)	− 1.3 (1.6)	− 1.9 (1.6)	**0.04**
“Bleeding as usual”	240 (88%)	223 (97%)	17 (43%)	**< 0.01**
“Bleeding more than usual”	30 (12%)	7 (3%)	23 (57%)	

Mean (*SD* standard deviation) or number (%) as appropriate. Statistical significance (*p* < 0.05) is highlighted in bold. ΔHb (g/dl) (= post-op Hb (g/dl)–pre-op Hb (g/dl))

**Fig. 1:**
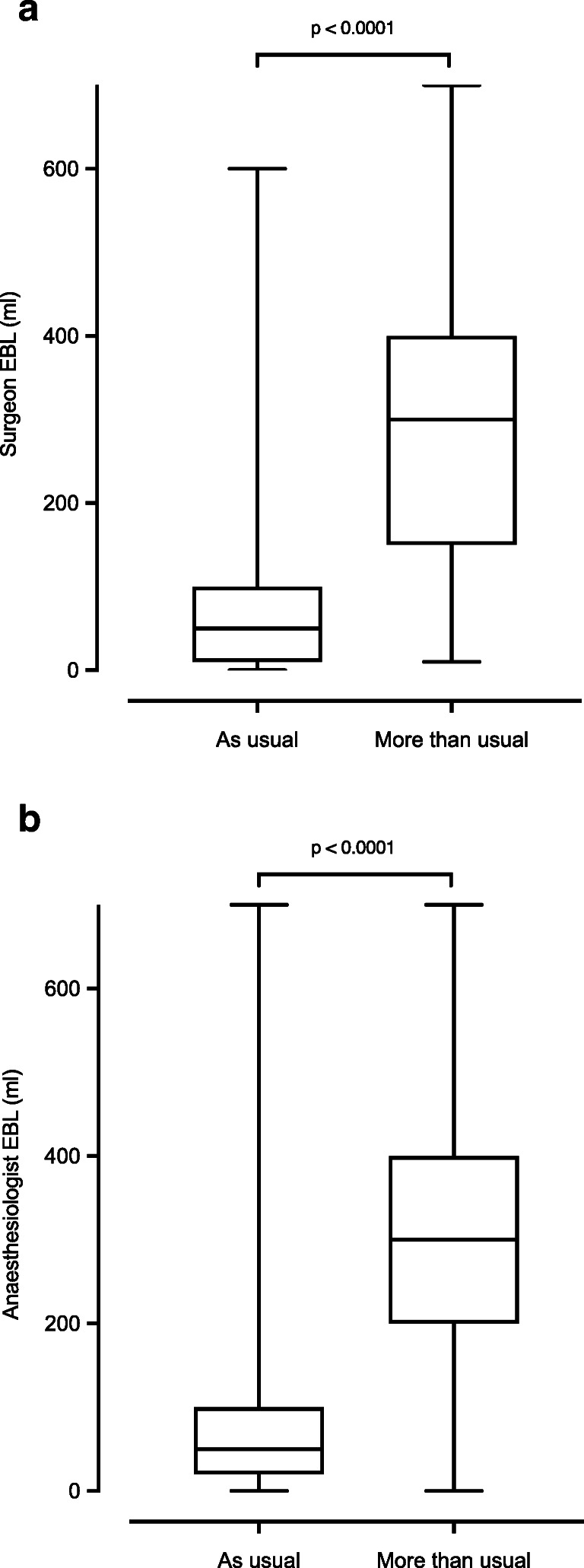
**a**, **b** EBL (ml) comparison between “usual” vs “more than usual” for surgeon (**a**) and anaesthesiologist (**b**)

Patients with a surgeon EBL ≥ 250 ml had more severe complications (20% vs. 8%, *p* = 0.02). Surgeon and anaesthesiologist mean EBL (ml) levels were lower in “usual” bleeding compared to “more than usual” bleeding (60 ml, SD 77 vs 297 ml, SD 149, *p* < 0.001 and 65.9 ml, SD 84 vs 312.6 ml, SD 185, *p* < 0.001, Fig. 1a, b). Patients with “more than usual” bleeding had significantly more severe complications (27% vs. 8%, *p* = 0.001), higher mortality (11% vs. 1%, *p* = 0.001) and longer length of stay (21 vs 12 days, *p* = 0.004, Tables 3 and 4).

**Table 3:** Post-operative complication and length of stay: comparison of surgeon EBL < 250 ml vs ≥ 250 ml

	EBL < 250 ml *n* = 230	EBL ≥ 250 ml *n* = 40	*p* value
Overall complications	71 (31%)	15 (38%)	0.4
Post-operative bleeding*	15 (7%)	4 (10%)	0.6
Severe complications	19 (8%)	8 (20%)	**0.02**
30 days mortality	4 (1%)	2 (5%)	0.2
Mean LoS (days)	13 (13)	17 (16)	0.09

Mean (*SD* standard deviation) or number (%) as appropriate. LoS: length of stay in days. Statistical significance (*p* < 0.05) is highlighted in bold. Complications according to Dindo-Clavien classification [1]. Post-operative bleeding: complication defined as grade 2 or more (needed at least one transfusion) according to Dindo-Clavien

**Table 4:** Post-operative complication and length of stay: comparison of bleeding as expected vs bleeding more than expected

	As usual *n* = 240	More than usual *n* = 30	*p* value
Overall complications	74 (31%)	12 (40%)	0.3
Post-operative bleeding*	15 (6%)	4 (13%)	0.2
Severe complications	19 (8%)	8 (27%)	**0.001**
30 days mortality	3 (1%)	3 (10%)	**0.001**
Mean LoS (days)	12 (12)	21 (19)	**0.004**

Mean (*SD* standard deviation) or number (%) as appropriate. LoS: length of stay in days. Statistical significance (*p* < 0.05) is highlighted in bold. Complications according to Dindo-Clavien classification [1]. Post-operative bleeding: complication defined as grade 2 or more (needed at least one transfusion) according to Dindo-Clavien

**Fig. 2:**
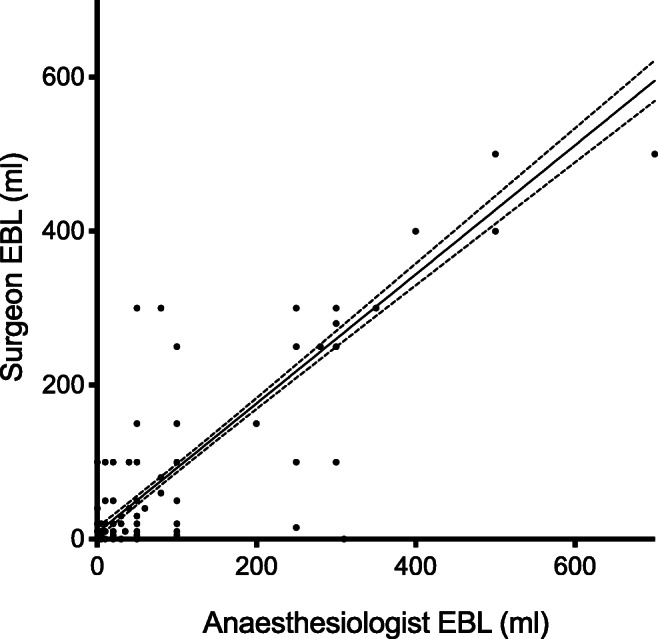
Correlation between surgeon EBL (ml) vs anaesthesiologist EBL (ml). Caption: ϱ = 0.9259, *p* < 0.0001

**Fig. 3:**
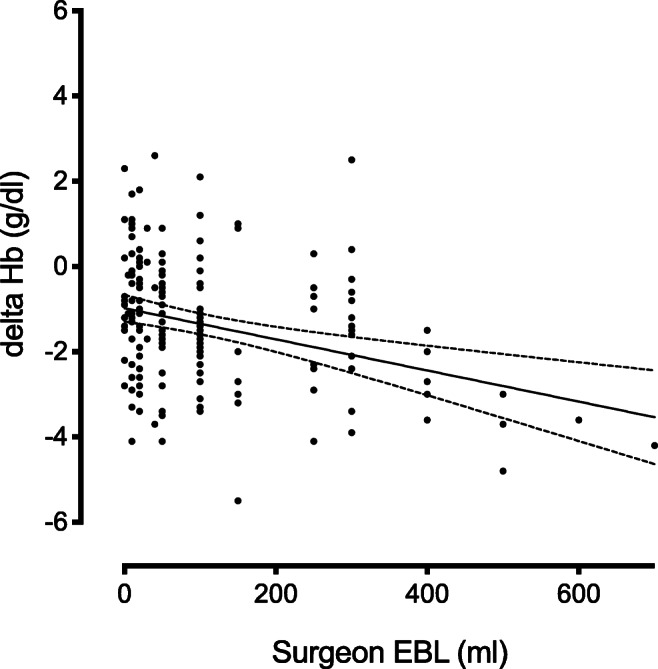
Correlation between surgeon EBL (ml) and difference between pre- and post-operative Hb values (g/dl) (delta Hb). Caption: ϱ = − 0.2730, *p* = 0.01

There was no difference between mean EBL documented by surgeons and anaesthesiologist (79.5 ml, SD 99 vs 84.5 ml, SD 118, *p* = 0.57). Significant correlation between surgeon and anaesthesiologist EBL was observed (ϱ = 0.926, *p* < 0.0001, Fig. 2), and surgeon EBL was correlated to ΔHb (ϱ = − 0.273, *p* = 0.01, Fig. 3). A correlation between anaesthesiologist EBL and ΔHb was also observed (ϱ = − 0.344, *p* = 0.01).

## Discussion

No difference between anaesthesiologist and surgeon’s EBL was observed in this study, and EBL correlated with ΔHb. If blood loss was considered “more than usual” by the surgeon, more severe complications, higher mortality rates and longer length of stay were observed.

There is no gold standard reference for recording intraoperative blood loss. However, EBL is reported in nearly all surgical technical papers as a precious, simple postoperative data allowing the identification of patients at risk of postoperative complications before they reach the transfusion threshold [9–13]. The association between blood loss and post-operative complications has been reported in colorectal surgery [2, 4, 5, 14], as well as in a range of specialties, including hepatic, gastric and cardiothoracic surgery [15–17]. Furthermore, EBL is used in predictive scores of post-operative adverse events in surgery, such as P-POSSUM [18, 19]. However, estimation of blood loss for a surgical procedure is both poorly reproducible and typically underestimated [20]. Measurement and interpretation of EBL are currently not standardized [20–26]. Despite studies pointing that EBL is not a precise tool, others highlight that accuracy significantly improves with specific training [20–26]. For example Stahl et al. proposed a tool based on the haemoglobin level at 24 and 48 h [24]. Rothermel et al. concluded that visual estimation of operative blood loss was unreliable and inaccurate, and that measurement of the suction liquids added to the weight of gauzes was a better method considered [22]. Ultrasound of the inferior vena cava, contrast enhanced ultrasound, near-infrared spectroscopy, continuous non-invasive intraoperative Hb monitoring or gravimetric and colorimetric measurements have also been described [27]. In the present study, EBL measured by the anaesthesiologists was based on the suction liquid from which they subtract the flushing liquid. Blood loss estimation made by the surgeon was visual. No differences were displayed between the two observers regarding EBL in volume (ml). Surgeon EBL correlated well with postoperative haemoglobin drop, which may suggest a reliability of the surgeon's estimation.

There is a great heterogeneity of EBL cut-off values and influence on postoperative complications. For example, eight studies reported the influence of blood loss on the rate of anastomotic leaks [4, 14, 28–33]. Two studies [14] concluded that > 300 ml EBL was significantly associated with the risk of anastomotic leak after colectomy while others reported cut-off values > 250 ml [4], > 200 ml [31] to be significantly associated with anastomotic leak. In rectal cancer surgery, even larger volumes were reported (> 1500 ml [30] and > 4500 ml [29]). McGillicudy et al. [5] and Egenvall et al. [34] reported that blood loss > 1000 ml and > 450 ml, respectively, was significantly associated with mortality. Volume of EBL in ml and their impact on outcome vary greatly.

In the present study, EBL greater or equal to 250 ml correlated to post-operative complications but did not correlate to post-operative mortality. A simple binary EBL grading correlated with both 30-day postoperative complications and mortality. Looking at the correlation between objective blood loss and subjective binary estimation, it is obvious that EBL < 250 ml seems to be "usual" for most surgeons. Interestingly, EBL of > 250 ml seems to trigger the assessment "more than usual" in only about half of the cases in the present study. Therefore, the trigger to assess blood loss as "more than usual" seems to be higher than 250 ml for most surgeons. This could explain the higher correlation between morbidity and mortality of the binary EBL in comparison with measured EBL. However, complex and non-validated measurement methods that have been described for EBL could be avoided and replaced by this completely subjective and binary measure, which is equivalent to estimation in volume but much easier and faster to apply in surgeon’s daily practice. This would allow to predict postoperative outcomes and thus adapt management with the aim of improving patient outcome. It could also be considered to indicate the need for early postoperative transfusions. However, this needs to be demonstrated in well-designed large-scale study.

Several limitations of the present study need to be addressed. First, the relatively small sample size and the retrospective analyses could both affect the quality of the data. However, all values were collected prospectively, as well as the binary grading of EBL. Furthermore, it cannot be excluded that surgeons and anaesthesiologist agreed on the volume (ml) at the end of the operation even if they documented it separately. There was, however, no systematic communication between the surgeon and the anaesthesiologists on EBL and both could have over- or under-estimate intraoperative blood loss. Indeed, only one anaesthesiologist and one surgeon completed the form, and no inter-rater reliability (Cohen’s kappa coefficient) was done. Another point to consider is the fact that the subjective evaluation could represent the postoperative state of mind of the surgeon, and instinctively, one would tend to overestimate the bleeding if the surgery is complex, thus inserting potential bias. Otherwise, changes in Hb levels are dependent on the hydration of the patient and the volume of fluids administered during surgery. In this study, this aspect was not considered which could have induced bias. As previously described, clinically relevant EBL cut-off was set at 250 ml [4, 6], but the results could have varied if another cut-off had been chosen. In addition, after long and stressful procedures, surgeons may tend to overestimate the blood loss (“more than usual”), and due to the increased fluid volumes, the resulting ΔHb may support the value of the binary EBL. Depending on the patient (comorbidities, coagulation disorders, preoperative anaemia) or the surgeon, this subjective estimation may vary. Further investigations are required to see if it is reproducible and generalizable on a large scale.

## Conclusion

In conclusion, there was no difference between anaesthesiologist and surgeon’s EBL. If blood loss was considered “more than usual” by the surgeon, more severe complications, higher mortality rates and longer length of stay were observed. Thus, EBL in volume did not appear superior to simple binary subjective estimation from the surgeon, which was reliable in predicting postoperative outcomes in colon surgery.
